# Genome analysis following a national increase in Scarlet Fever in England 2014

**DOI:** 10.1186/s12864-017-3603-z

**Published:** 2017-03-10

**Authors:** Victoria Chalker, Aleksey Jironkin, Juliana Coelho, Ali Al-Shahib, Steve Platt, Georgia Kapatai, Roger Daniel, Chenchal Dhami, Marisa Laranjeira, Timothy Chambers, Rebecca Guy, Theresa Lamagni, Timothy Harrison, Meera Chand, Alan P. Johnson, Anthony Underwood, The Scarlet Fever Incident Management Team

**Affiliations:** 1 National Infection Service, Public Health England, 61 Colindale Avenue, London, NW9 5HT UK; 2 Guy’s and St Thomas’ NHS Foundation Trust, London, UK

**Keywords:** Scarlet Fever, *Streptococcus pyogenes*, Genomics, iGAS, Invasive GAS

## Abstract

**Background:**

During a substantial elevation in scarlet fever (SF) notifications in 2014 a national genomic study was undertaken of *Streptococcus pyogenes* (Group A Streptococci, GAS) isolates from patients with SF with comparison to isolates from patients with invasive disease (iGAS) to test the hypotheses that the increase in SF was due to either the introduction of one or more new/emerging strains in the population in England or the transmission of a known genetic element through the population of GAS by horizontal gene transfer (HGT) resulting in infections with an increased likelihood of causing SF. Isolates were collected to provide geographical representation, for approximately 5% SF isolates from each region from 1^st^ April 2014 to 18^th^ June 2014. Contemporaneous iGAS isolates for which genomic data were available were included for comparison. Data were analysed in order to determine *emm* gene sequence type, phylogenetic lineage and genomic clade representation, the presence of known prophage elements and the presence of genes known to confer pathogenicity and resistance to antibiotics.

**Results:**

555 isolates were analysed, 303 from patients with SF and 252 from patients with iGAS. Isolates from patients with SF were of multiple distinct *emm* sequence types and phylogenetic lineages. Prior to data normalisation, *emm*3 was the predominant type (accounting for 42.9% of SF isolates, 130/303 95%CI 37.5–48.5; 14.7% higher than the percentage of *emm*3 isolates found in the iGAS isolates). Post-normalisation *emm* types, 4 and 12, were found to be over-represented in patients with SF versus iGAS (*p* < 0.001). A single gene, *ssa,* was over-represented in isolates from patients with SF. No single phage was found to be over represented in SF vs iGAS. However, a “meta-ssa” phage defined by the presence of :315.2, SPsP6, MGAS10750.3 or HK360ssa, was found to be over represented. The HKU360.vir phage was not detected yet the HKU360.ssa phage was present in 43/63 *emm*12 isolates but not found to be over-represented in isolates from patients with SF.

**Conclusions:**

There is no evidence that the increased number of SF cases was a strain-specific or known mobile element specific phenomenon, as the increase in SF cases was associated with multiple lineages of GAS.

**Electronic supplementary material:**

The online version of this article (doi:10.1186/s12864-017-3603-z) contains supplementary material, which is available to authorized users.

## Background

*Streptococcus pyogenes* (Lancefield group A streptococcus, GAS) is a common cause of bacterial throat infections, and also causes mild to severe skin and soft tissue infections, including impetigo, erysipelas and necrotizing fasciitis. GAS can also cause systemic infections including septicaemia and meningitis, which can be fatal. Estimated annual incidence for invasive GAS (iGAS) infection in industrialised countries is approximately 3 per 100,000 per year [1]. Scarlet fever (SF) is characterised by rash, “strawberry tongue”, and exudative pharyngitis and is thought to be due to infection with GAS secreting superantigens [2]. SF typically has a cyclical incidence, with resurgences occurring on average every 4 years [3]. In England and Wales the incidence of SF has been in decline for several decades, reducing from 250 notifications per 100,000 population per year in 1944 to <5 per 100,000 per year in the 2000s, until the marked increase in 2013/2014 season for which 25 notifications per 100,000 were noted in the 2013/2014 season [4, 5]. The 2013/14 routine surveillance of notifiable infectious diseases showed an unprecedented increase in the number of cases of SF despite iGAS numbers remaining within expected seasonal range [6]. A study in the North-West London indicated and association of SF with *emm*3 lineage isolates and an increase in *emm*3 and *emm*4 infections co-incident with the upsurge [7]. Previous studies have shown an over-representation of *ssa, speA* and *speC* in SF isolates [8]. Similarly, multiple *emm*1 SF isolates were recently noted in Hong Kong encoding SSA [2]. In England, a novel phage has been described in *emm*3 lineage invasive and non-invasive GAS strains encoding SpeC [9]. Previous studies have also shown that in epidemic periods specific *emm* gene sequence types may predominate within the mixed population of strains present including in Taiwan (1998–2007) [10], Hong Kong [11] and also in a study of pharyngeal isolates from patients with and without SF in Lisbon 2002–2008 [8]. Due to the unprecedented increase in the incidence of SF in England and Wales, a national incident management team was convened by PHE which initiated investigation of isolates of GAS from patients with SF, with the intention of collating a representative selection of SF isolates from all regions of England for genomic analysis. This was undertaken to determine whether the increase in SF was due to the introduction of new/emerging strain/s into the population or transmission of a known genetic element through the population of GAS by horizontal gene transfer (HGT) resulting in infections with an increased likelihood of causing SF.

## Results

A total of 555 isolates of which 303 were from SF patients and 252 from iGAS patients were included in the analysis.

### *emm* gene typing

**Fig. 1:**
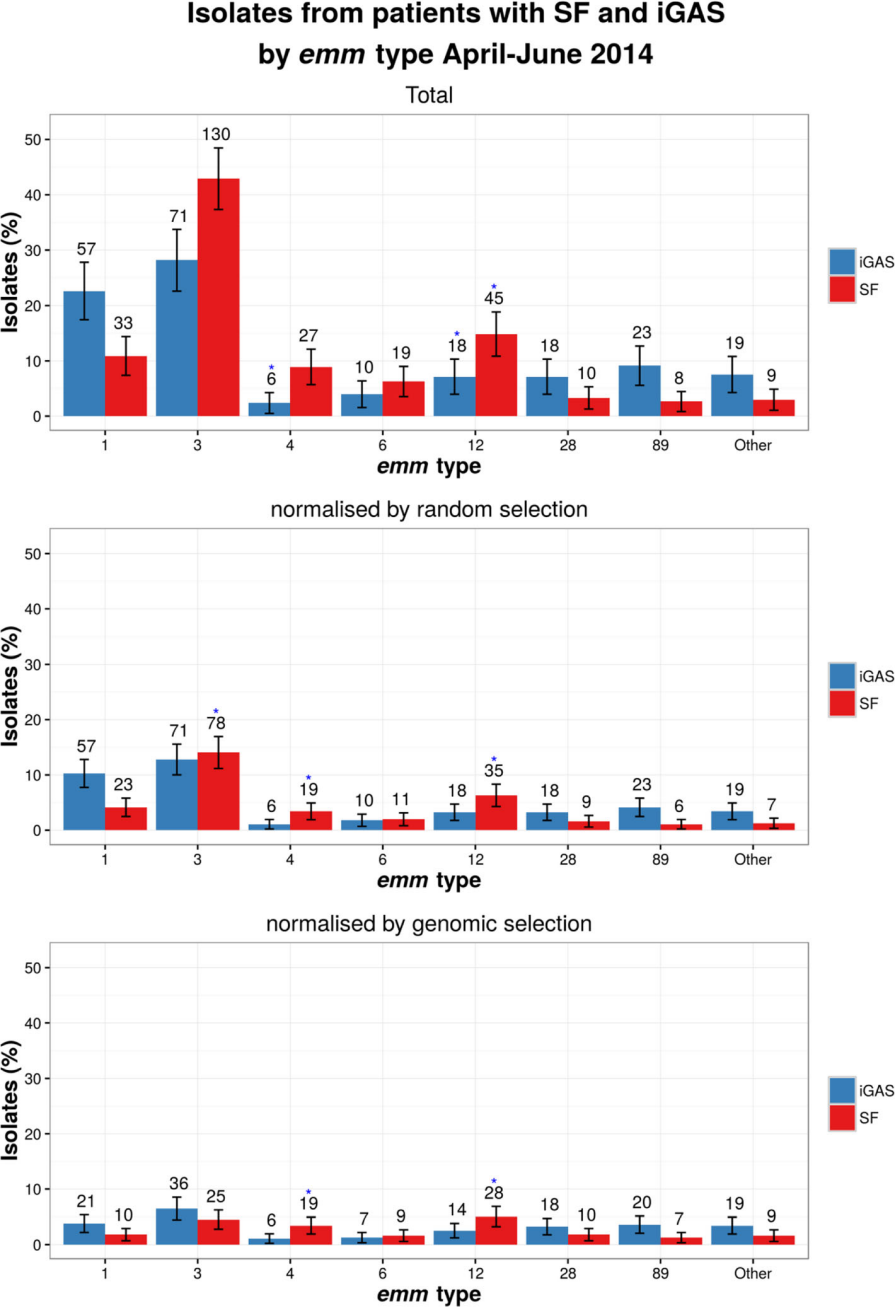
*emm* gene type in patients with SF and iGAS. *emm* types detected in > 5% population of patients with SF and iGAS (note *emm* 4 and 6 were <5% for iGAS and are included for comparison to SF). Note the total isolate number per *emm* type and for all isolates and normalised random and genomic selection is included, in addition to percentage, 95% confidence intervals and the p value obtained using Fisher’s exact test (with Bonferroni’s correction) to compare number of isolates per emm type per study selection groups versus iGAS isolates (*blue star* indicates *p* = <0.05)

Analysis of 555 isolates in total including 303 from patients with SF and 252 from patients with iGAS indicated 16 *emm* types (*emm* 1, 2, 3, 4, 6, 9, 12, 22, 28, 44, 58, 75, 77, 82, 87 and 89) in the study population from patients with SF, with *emm* 1, 3, 4, 6 and 12, each accounting for more than 5% of the population (Fig. 1 and Additional file 1). Isolates of 26 *emm* types (*emm* 1, 2, 3, 4, 5, 6, 9, 12, 18, 22, 28, 44, 53, 58, 73, 75, 76, 77, 81, 82, 87,89, 90, 94, 103 and 171 were identified among the 252 isolates of patients with iGAS, with *emm* 1, 3, 12, 28, and 89 each accounting for more than 5% of the population (Fig. 1 and Additional file 1). Isolates from patients with SF were of multiple distinct *emm* sequence types with *emm*3 predominating, accounting for 42.9% (130/303 95%CI 37.5–48.5); this was 14.7% higher than the proportion seen amongst the iGAS isolates (Fig. 1 and Additional file 1). Although there appeared to be an association between *emm*3 and SF phenotype, this was not found to be statistically significant following application of Bonferoni’s correction, when sampling bias per submitting region (using the normalised random selection) was considered. This association was also not found to be significant when genomic lineage (normalised genomic selection) were taken into consideration (Fig. 1 and Additional file 1). Two *emm* types (*emm* types 4 and 12) were consistently found to be over-represented in isolates from patients with SF versus iGAS (*p* = 0.0008 and 0.0014, respectively) following application of Bonferroni’s correction.

### Phylogenetic analysis

**Fig. 2:**
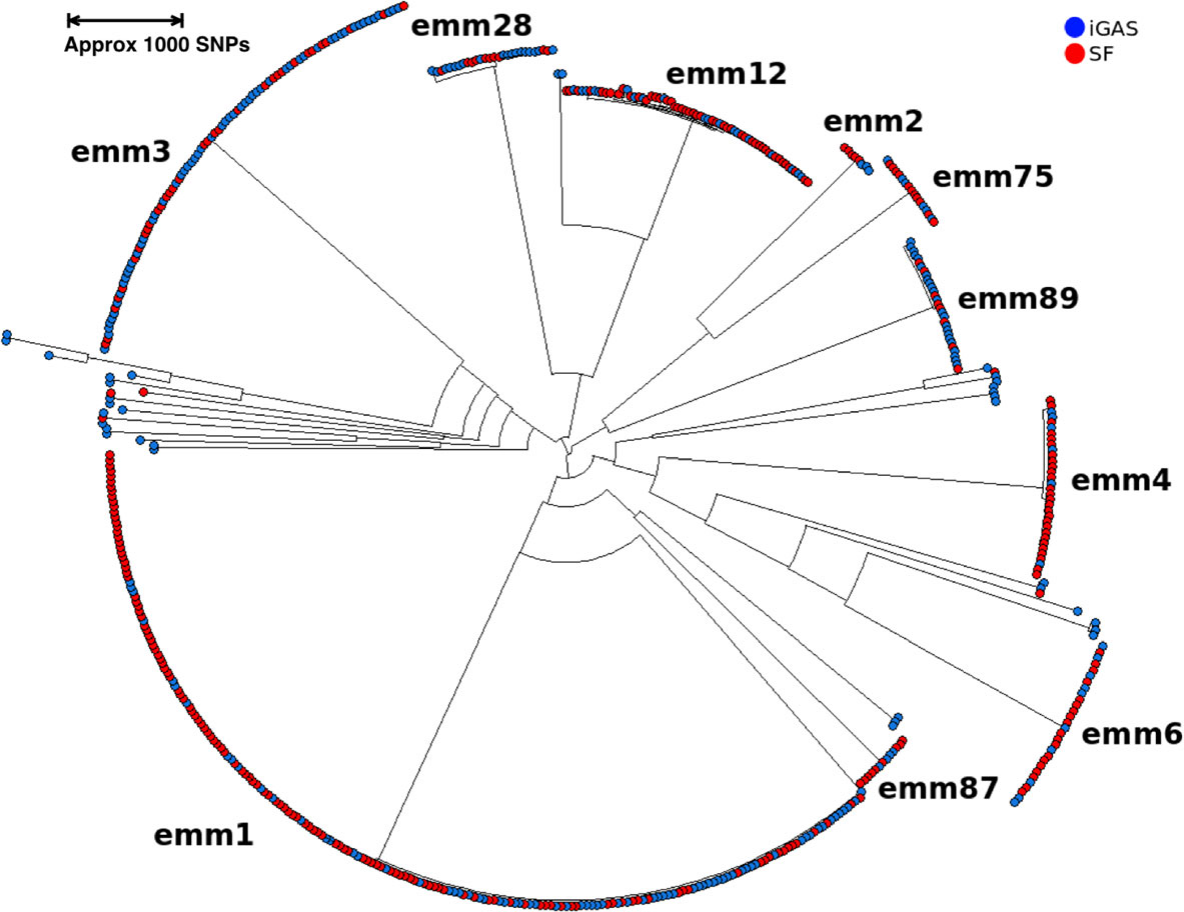
Maximum likelihood phylogenetic tree. Maximum likelihood phylogeny with *emm* type. RAxML phylogeny based on SNP variants of SF and iGAS mapped to MGAS2096. SF isolates are coloured *red* and iGAS isolates in *blue* (April to June) The radial branch lengths within the tree represent approximately 4000 SNPs

The population structure of SF and iGAS isolates (maximum likelihood) is depicted in Fig. 2. The radial branch lengths within the tree represent approximately 4000 SNPs. The iGAS and SF isolate sequences were found to be evenly spread throughout the phylogeny with no obvious association within a single or small number of clades. For comparison the percentage of historic referred isolates per *emm* type from patients with SF is included from 2004 to 2014 (Additional file 2).

### Population structure, hierarchical clustering

**Fig. 3:**
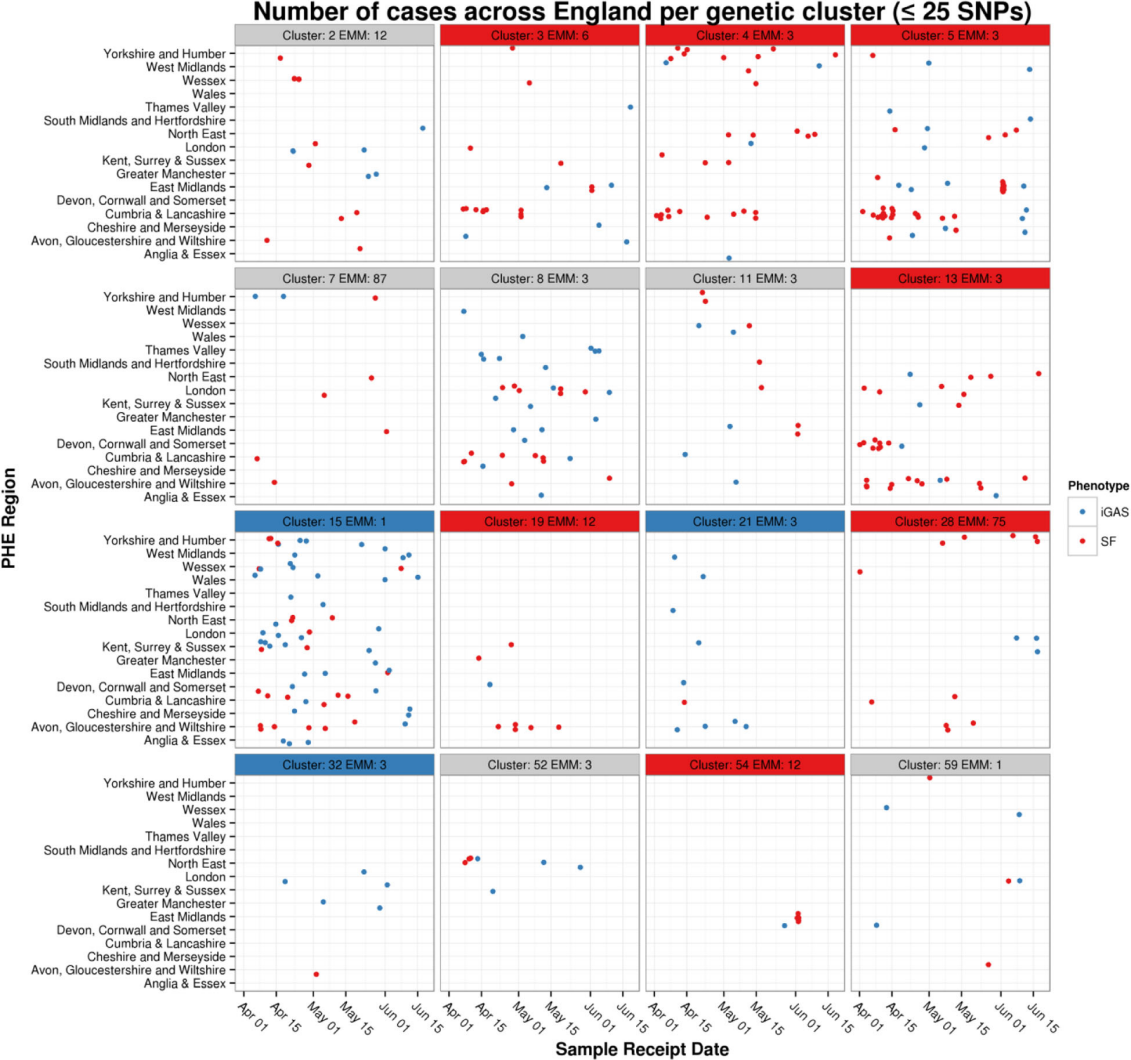
Genomic cluster (<26 SNP) per region and time. Sixteen genomic clades (April to June) with more than 5 referred isolates are plotted - the number in title boxes represent the arbitrary cluster number followed by the *emm* type. Those clusters significantly associated with iGAS and SF are coloured with *blue* and *red* headers respectively

Isolates were grouped into genomic clusters based on hierarchical clustering using the number of SNP differences as the distance metric and complete linkage at less than 26 SNP differences. 161 genomic clusters were identified, of which the majority had less than 5 isolates.. The Cochran-Mantel-Haenszel test was used (April to June) to look for association of genomic clusters with differing phenotypes, with results plotted by region and time (Fig. 3). Of the 16 with more than 5 isolates per genomic cluster using the Cochran-Mantel-Haenszel, 6 did not have over-representation of SF vs iGAS or vice versa, 7 were over-represented in SF isolates vs iGAS and 3 were over-represented in iGAS. When looking at specific genomic clusters (<26 SNP) per region over time (Fig. 3) all genetic clusters with more than 5 representative strains had isolates found in patients with iGAS or SF. In many (7/8) of the larger clusters with greater than 15 isolates (cluster numbers: 2, 3, 4, 5, 8, 11, 15 on Fig. 3) the same genomic SNP clusters were found to have representative isolates from patients with iGAS and SF distributed throughout the time period studied. In genetic clusters with smaller numbers (*n* < 15), uneven sampling will have had a greater impact on the distribution and this may account for the apparent less even distribution of iGAS and SF isolates over time in smaller clusters. Not all regions were able to provide sufficient SF isolates, with one not submitting any, and others submitted more than the 5% target (Additional file 3).

### Age and genomic cluster analysis

**Fig. 4:**
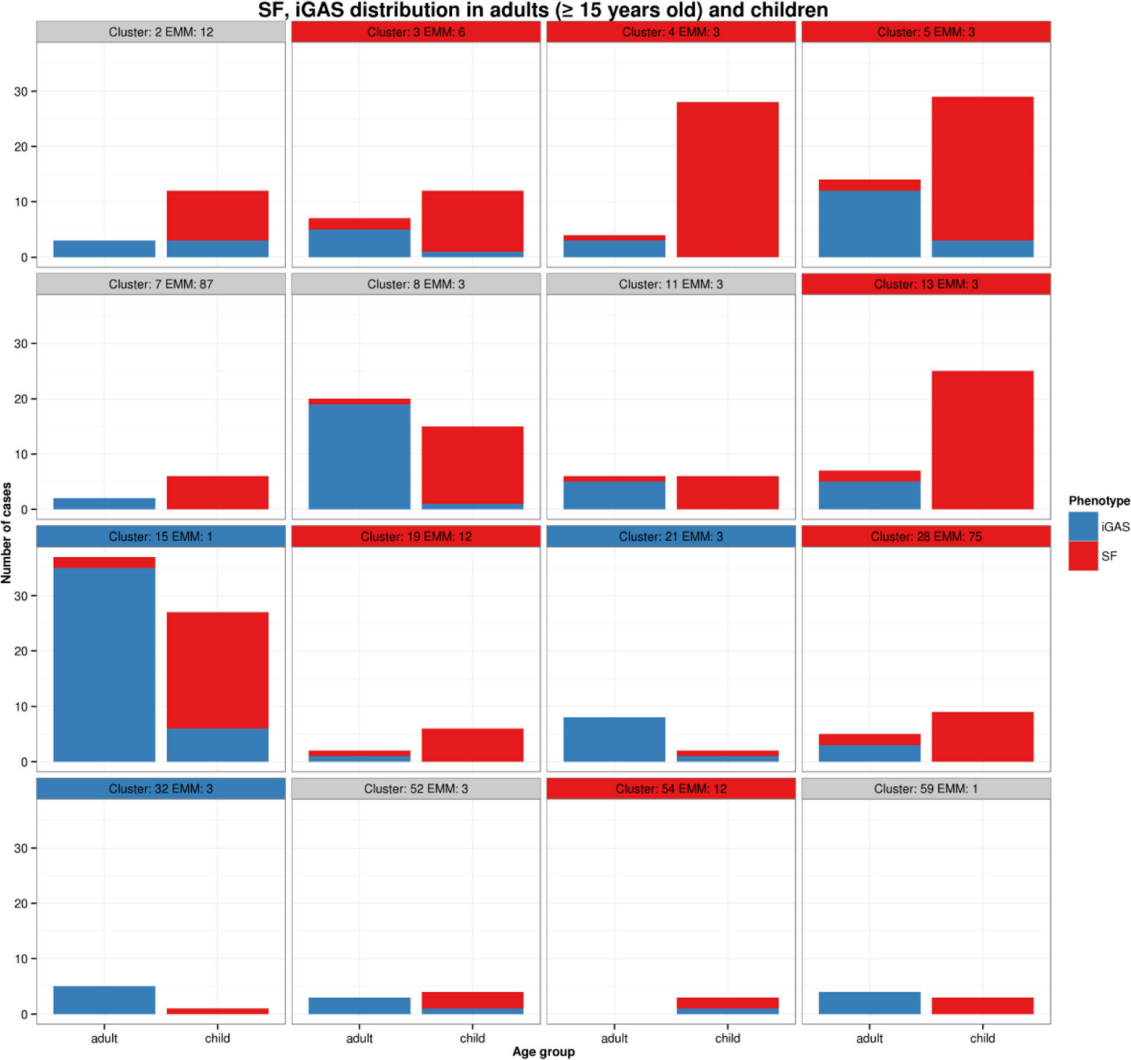
Age and genomic cluster. Strains of the same genomic clades were found in both adult and child populations. Strains within specific genomic clusters overrepresented in SF (*red title boxes*) were also found in adult patients with iGAS. Similarly, clusters over-represented in iGAS (*blue*) all contained cases of SF

All genomic clusters where SF isolates were over-represented also contained isolates from adults with iGAS, indicating absence of a specific strain lineage associated with SF (Fig. 4).

### Horizontal gene transfer (HGT)

Analysis of the association of genes with isolates from cases of SF versus iGAS showed that *ssa* was present in all SF isolates (Additional file 4). However, *ssa* was also found in isolates from patients with iGAS (using normalised random selection of isolates from the study period in 2014).

### Phage content

Of 57 phage sequences included in the study (Additional file 5) 54 were previously described within *emm* types 1, 2, 3, 4, 5, 6, 12, 18, 28 [12–14]. Examination of the subset of isolates that consisted of only these *emm* types (242 SF and 177 iGAS isolates) revealed that no phage was significantly associated with isolates from patients with SF. However, previous reports have linked presence of *ssa* gene with SF, We hypothesised that closely related phage may be carrying *ssa* gene, but may not be individually associated with the SF samples. To test this hypothesis, new “meta-ssa” phage was defined to be present if any one of the following phage were present: 315.2, SPsP6. MGAS10750.3 or HK360ssa. These phage have been characterised to contain *ssa* gene [11, 14, 15]. Repeating the phage association analysis with the additional phage showed association of the “meta-ssa” with SF (Fisher’s Exact Test *p* = 0.0000025, Bonferroni correction = 0.00014). Association of “meta-ssa” phage, but not indivdual *ssa* carrying phages points towards a polyclonal population of SF phage. Although not significantly over-represented in *emm* 12, isolates from patients with SF compared to those with iGAS, a phage with >95% identity to the HKU360.ssa phage {Davies, 2015 #18} was found in 43/63 (68.3%, 95% CI 55.–78.5) *emm*12 isolates and 1/28 (3.5%, 95% CI 0.01–19.2) of *emm*28 isolates, and were absent from all other isolates in this study.

## Discussion

Due to the unprecedented increase in the incidence of SF in England and Wales in 2014 genomic analysis was undertaken to determine whether the increase in SF was due to the introduction of new/emerging strain/s into the population or transmission of a known genetic element through the population of GAS by HGT resulting in infections with an increased likelihood of causing SF. This study found no evidence to support the hypothesis that the increased number of cases of SF was due to the introduction of, or increase in, a single genetic lineage within the population or to a known virulence gene/phage phenomenon as analysis of the population structure (Fig. 2) showed that isolates belonging to multiple diverse genetic lineages were present in patients with SF. Isolates from the same lineages were also present in patients with iGAS. Further analysis revealed seven defined genomic clusters (<26 SNPs) with >5 isolates; seven over-represented in patients with SF versus iGAS, and three in iGAS versus SF. However, SF and iGAS isolates were found in all genomic clusters with >5 isolates. It is possible that isolates within these clusters are more commonly carried in the throat or have greater propensity to cause invasive infection in humans than other lineage strains. However, the prevalence of these lineages in asymptomatic individuals is not known. Nonetheless, all 16 clusters were found in differing geographical areas and across the time period studied, indicating widespread presence in the population. Studies have shown that an expansion of an iGAS clone from patients in the San Francisco Bay area during 2005–2008 differed on average by only 10 SNPs within 14 isolates [16] The phylogenetic and epidemiologic data suggest that these 14 isolates constitute a distinct clone that caused a geographic cluster of invasive infections [16, 17]. Therefore, the variation observed within the isolates in <26 SNP clusters included in this study must have arisen over a long time period and not within the documented period of increase in SF in 2014.

In 2009 a rise was noted in SF in Vietnam [referenced in [18], and in 2011 increases of SF were reported in China and Hong Kong [2, 11, 18, 19]. These increases were attributed to mixed *emm* lineage clones with multiple clones of *emm*12 predominating, followed by *emm*1 including strains in which an integrative conjugative element encoding resistance to tetracycline (TetM), erythromycin (ErmB) and clindamycin, and bacteriophage HKU488.vir encoding superantigens SSA and SpeC were noted [2, 17]. The finding from this study that the *ssa* gene was significantly associated with isolates from patients with SF is not surprising, as this is consistent with findings reported by other smaller studies [2, 8]. It has been speculated that in a single *emm* lineage the acquisition of *ssa* can confer potential changes in disease and pathogenicity [2]. However this has not been demonstrated in multiple lineages, and is unlikely to occur within an entire population of multiple lineages within the limited time period of a few months. In terms of phage, no single known phage was found in this study, rather several differing phage carrying *ssa* in isolates from patients with SF were noted. However, strains were not analysed for as yet undiscovered phage harbouring *ssa* or additional genetic factors, hence such genetic elements cannot be fully discounted.

In this study several *emm* GAS lineages were found in patients with SF, similar to that seen historically in England (Additional file 2). Two *emm* lineage clusters were found to be over-represented in SF isolates versus iGAS; *emm*4 and *emm*12, consistent with findings from North West London of an over-representation of *emm*4 [7]. These two *emm* lineages were not found within the same phylogenetic major clade (Fig. 2). All published data regarding large increases in wide geographic areas in SF have involved multiple *emm* lineage strains, albeit with a single predominant *emm* lineage described for each study, such as *emm*12 in Taiwan, China and Hong Kong in 2011 [10, 11, 19]. The predominant strain *emm* lineage in this study was also found to be *emm*3. However this lineage was not significantly over-represented in isolates from patients with SF versus iGAS, unlike Turner et al. [7] comparison to isolates from the upper respiratory tract. Historical submissions of isolates from 2004 to 2015 from patients with SF indicate an annual fluctuation in the predominant *emm* type strain *per annum*, including *emm*3, 4 and 12 and could indicate type specific fluctuation in population immunity (Additional file 2). However these isolates are submitted on an ad hoc basis and are not from a defined sampling scheme. Changes in predominant *emm* types in children with SF in the Beijing population over time and with area specific changes have recently been documented [20].

Further study of two genetic elements recently discovered within some *emm*12 lineage strains (the integrative and conjugative element ICE-*emm*12, encoding genes for tetracycline and macrolide resistance, and prophage ΦHKU.vir, encoding the superantigens SSA and SpeC, as well as the DNase Spd1) may uncover an important role for these elements in conferring the SF phenotype in patients [2, 11]. The HKU360.vir phage described in some isolates from the 2011 Hong Kong SF increase [11] was not present in isolates in this study, whereas the HKU360.ssa was present in the majority of *emm*12 type isolates but was not found to be over-represented in isolates from patients with SF. In summary, the large observed increase in the number of SF cases cannot be attributed to a single genetic factor. Without being able to determine the proportion of the different lineages and the genetic factors they carry within the asymptomatic/carriages population it is not possible to determine if the lineages observed in the disease causing isolates mirrors those seen in the population as whole. If the population structure within these two human host groups was similar it would lend weight to alternative hypotheses such as the increase in SF cases being due to alternative factors. These could include host population immunity status, concurrent circulation of other predisposing pathogens such as respiratory viruses, potential declining healthcare attendance for patients with GAS infection resulting in increased likelihood for infection to progress to SF and reduction in the use of antimicrobials affecting pharyngeal carriage in the population.

Geographically representative sampling was limited by isolates available in clinical laboratories and consequently 9 of 15 regions of England submitted fewer samples than the requested 5% sample and multiple submissions from localised clusters could not be discounted during collection. Isolates from periods outside the study period, including those during the height of the epidemic peak were not available for comparison. Therefore sufficient isolates were not available to undertake a comprehensive genome wide association study to determine additional other/novel genetic factors associated with SF.

## Conclusions

The increase in SF cases was not caused by the introduction of a single or dominant clone but is associated with multiple lineages within the GAS population currently circulating in England. The *ssa* gene was over-represented in isolates from patients with SF. Although no single *ssa* carrying phage was found to be over represented in SF vs iGAS, the “meta-ssa” phage defined by the presence of :315.2, SPsP6. MGAS10750.3 or HK360ssa (phages previously shown to contain *ssa*, Additional file 5), was found to be over represented in isolates from patients with SF. The HKU360.vir phage described in some isolates from the 2011 Hong Kong SF increase [11] was not present in any of the 555 isolates analysed in this study. The HKU360.ssa phage associated with some *emm*12 isolates from the 2011 Hong Kong SF study was present in 43/63 *emm12* isolates, but was not found to be over-represented in isolates from patients with SF. Two *emm* types, 4 and 12, were found to be over-represented in patients with SF versus iGAS (*p* = 0.0008 and *p* = 0.0014 respectively), but were not restricted to SF patients. The *emm*3 lineage isolates were not found to be over-represented following genomic and regional normalisation indicating a large proportion of *emm*3 isolates were received from a single region. Other factors need to be examined to determine contribution to the national reported increase in SF.

## Methods

### Aim, design and setting

This genomic study was undertaken to determine whether the increase in SF in England 2014 was due to the introduction of new/emerging strain/s into the population or transmission of a known genetic element through the population of GAS by horizontal gene transfer (HGT) resulting in infections with an increased likelihood of causing SF.

### Strain collection

In an attempt to assemble a representative sample of circulating GAS isolates from SF a request was made to microbiology laboratories in England to submit routinely obtained isolates of GAS to PHE Colindale. A sampling scheme was developed based on number of SF notifications per region with each requested to supply a target number of isolates equivalent to ~5% of SF notifications from each of the 15 regions, for specimens taken between 1^st^ April 2014 and 18^th^ June 2014 submitted to the National Reference Laboratory. A total of 15 regions were defined as follows: Anglia & Essex; Avon, Gloucestershire & Wiltshire; Cheshire & Merseyside; Cumbria & Lancashire; Devon, Cornwall & Somerset; East Midlands; Greater Manchester; Kent, Surrey & Sussex; London; North East; South Midlands & Hertfordshire; Thames Valley; Wessex; West Midlands; Yorkshire & Humber. Not all regions were able to provide sufficient SF isolates, with one not submitting any, and others submitted more than the 5% target (Additional file 3). A control groups comprised of contemporaneous iGAS isolates routinely submitted as part of ongoing national surveillance, for which genomic data were already available, were included in the analysis. A total of 555 isolates of which 303 were from SF patients and 252 from iGAS patients were included in the analysis. Isolates received outside of the target dates were excluded from this analysis.

### Genomic sequencing (GS)

Genomic DNA was extracted from 5 to 6 colonies per isolate after 24 h incubation at 37 °C on 5% horse blood agar (PHE media services) using the Qiasymphony (Qiasymphony DNA Mini kit, Qiagen) after after pre-lysis at 37 °C for 30 min and 100 °C for 10 min with 30 units mutanolysin and 0.06 mg hyaluronisdae (Sigma-Aldridch). Paired end multiplex libraries were created with the Illumina Nextera XT sample preparation kit, followed by sequencing on Illumina Hi-Seq 2500, with a read length of 2x100 bp. The Illumina sequence short read FASTQ files from all isolates were trimmed for quality by removing leading and trailing nucleotides of Phred quality score Q < 30, truncating a read if a sliding window of size 4 has a mean Q < 30, and dropping a whole read if shorter than 50 nucleotides after trimming.

### Analysis of association of *emm* type with phenotype

The *emm* gene type of all isolates was determined from genomic sequence (GS) data. Over-representation of *emm* gene type in isolates from patients with SF versus iGAS was examined using Fisher’s Exact test with Bonferroni’s correction (R statistics package). A limited number of additional strains of GAS were included from other periods or locations for comparison including the *emm*12 lineage strain reported in Hong Kong [11] and an *emm*3 lineage strain from a period of enhanced surveillance in the UK from 2008–2009 [9].

### Phylogenetic analysis, tree generation

Reads were mapped to the NCBI reference genome MGAS2096 (*emm*12) using bwa. Variants were called using GATK 2.6.5 and parsed to retain high quality single nucleotide polymorphisms (SNPs) based on the following conditions: depth of coverage (DP) ≥5, AD ratio (ratio between variant base and alternative bases) ≥0.8, Mapping Quality (MQ) ≥30, ratio of reads with MQ0 to total number of reads ≤0.05. All positions that fulfilled the filtering criteria in >0.9 of the samples were joined to produce a multiple fasta format file where the sequence for each strain consists of the concatenated variants. This file was used as an input to generate a maximum likelihood (ML) tree using RAxML [21] as implemented on the CIPRES portal [22] with the following parameters –m (substitutionModel) GTRCAT –b (bootstrapRandomNumberSeed) 12345 -# (numberOfRuns) 100 –c (numberOfCategories) 25.

### Phylogenetic analysis, population structure

The population structure of isolates was analysed by grouping them into genomic clusters based on complete linkage hierarchical clustering using the number of SNP differences as the distance metric and a threshold of 25. The Cochran-Mantel-Haenszel test was used to look for genomic clusters associated with cases of SF and iGAS.

### Isolate subset normalisation

In order to account for tests across multiple regions that may have had different data collection/sending strategies, as well as, multiple samples sent from the same outbreak, analysis was performed on three isolate subsets (isolate numbers per group and region are listed Additional file 3):

1. All isolates included in the study (total selection, stratified by region if more than 1 isolate was submitted from the region).
2. For regions where more than 5% of total isolates in the region were submitted, isolates were downsampled to the target 5% using random selection, while maintaining the proportion of observed *emm* types. For the regions where 5% of total cases in the region or less were submitted, no downsampling was performed (normalised random selection).
3. To control for inclusion of multiple isolates submitted from the same outbreak, all isolates were clustered using hierarhical clustering with complete linkage on SNP differences between isolates to create genetic SNP clustering. For clusters (<26 SNPs) with multiple isolates per region, a random isolate was picked to be “representative” of the region. This process (normalised genomic selection) was performed for both iGAS and SF isolates.

### Gene/Accessory genome analysis/Horizontal gene transfer (HGT)

To determine if HGT of specific genetic elements into isolates with different genetic backgrounds had occurred, the presence and absence of genes encoding known virulence factors and antibiotic resistance markers were determined (Additional file 4). The reads from GS were mapped to the sequences of known virulence factors (including superantigens and antibiotic resistance genes) and the resulting bam files parsed to find which genes/alleles were present in each isolate. This procedure was carried out using the GeneFinder software; an in-house algorithm that uses bowtie2 to map sequence reads to reference sequences of interest and Samtools vs 0.1.18 to genenerate an mpileup file, which is then parsed for the rapid detection of sought sequences [23]. Genes showing >90% nucleotide identity over the full length of their sequences were considered to be present. Accessory gene content in isolates from patients with SF and iGAS was examined.

Strains in genomic clusters comprising >5 strains were examined for the presence of 57 known phage sequences (Additional file 5) using GeneFinder and their association with SF isolates examined using a Fisher’s Exact test with Bonferonni’s correction If reads from an isolate mapped to a prophage reference sequence produced 95% overall nucleotide identity over the complete length of the phage, the phage was considered to be present in the isolate.

## Additional files

Additional file 1: Table of overrepresentation analysis of SF and iGAS. (DOC 49 kb)
Additional file 2: Figure of *emm* gene type reference laboratory referrals from patients with SF fever from 2004–2014. *emm* gene type reference laboratory referrals from patients with SF fever from 2004–2014. Note this dataset is based on referred isolates only and is therefore biased: RVPBRU collate SF isolates routinely if part of localised cluster investigation and numbers of referred isolates for SF isolates are low (95% CI not included). (DOC 212 kb)
Additional file 3: Table of isolate numbers per region. (DOC 44 kb)
Additional file 4: Table of known virulence factors and antibiotic resistance determinants examined in genomes. (DOC 35 kb)
Additional file 5: Table of 57 documented phage examined in genomes. (DOC 34 kb)

## Abbreviations

GAS: Lancefield group A streptococcus
HGT: Horizontal Gene Transfer
iGAS: invasive Group A Streptococci
ML: maximum likelihood
SF: Scarlet Fever
SNP: Single Nucleotide Polymorphism
