# Supplementary material for: Genome analysis following a national increase in Scarlet Fever in England 2014
Source: BMC Genomics. 2017 Mar 10;18:224. doi: 10.1186/s12864-017-3603-z (PMC5345146; doi:10.1186/s12864-017-3603-z)
Supplement: Additional file 1: — Table of overrepresentation analysis of SF and iGAS. (DOC 49 kb) [file 12864_2017_3603_MOESM1_ESM.doc]

**Additional file 1.** Table of overrepresentation analysis of SF and iGAS

|  |  | SF vs iGAS  Isolate number per emm type/Total Isolate number  % (95%CI)  Fisher’s exact test (with Bonferroni’s correction) p value | | |
| --- | --- | --- | --- | --- |
| SF/iGAS | *emm* type | All isolates in study | Normalised genomic selection | Normalised random selection |
| SF | 1 | 33/303  10.9 (7.8-14.9) | 10/132  7.6(4.0-13.5) | 23/202  11.4 (7.7-16.6) |
| iGAS |  | 57/252  22.6 (17.8-28.2) | 21/166  12.7 (8.4-18.6) | 57/252  22.6 (17.9-28.2) |
| P value |  | 0.99 (1) | 0.95 (1) | 0.99 (1) |
| SF | 3 | 130/303  42.9 (37.5-48.5) | 25/132  18.9 (13.1-26.5) | 78/202  38.6 (32.2-45.5) |
| iGAS |  | 71/252  28.2 (22.9-34.0) | 36/166  21.7 (16.1-28.6) | 71/252  28.2 (22.9-34.0) |
| P value |  | **0.0002 (0.006)** | 0.77 (1) | **0.01 (0.3)** |
| SF | 4 | 27/303  8.9 (6.2-12.7) | 19/132  14.4 (9.3-21.5) | 19/202  9.4 (6.0-14.3) |
| iGAS |  | 6/252  2.4 (0.9-5.2) | 6/166  3.6 (1.5-7.8) | 6/252  2.4 (1.0-5.2) |
| P value |  | **0.0008 (0.02)** | **0.0008 (0.02)** | **0.001 (0.03)** |
| SF | 6 | 19/303  6.3 (4.0-9.6) | 9/132  6.8 (3.5-12.6) | 11/202  5.5 (3.0-9.6) |
| iGAS |  | 10/252  3.9 (2.1-7.2) | 7/166  4.2 (1.9-8.6) | 10/252  4.0 (2.1-7.2) |
| P value |  | 0.2 (1) | 0.23 (1) | 0.3 (1) |
| SF | 12 | 45/303  14.9 (11.3-19.3) | 28/132  21.2 (15.0-29.0) | 35/202  17.3 (12.7-23.2) |
| iGAS |  | 18/252  7.1(4.5-11.1) | 14/166  8.4 (4.9-13.8) | 18/252  7.1 (4.5-11.1) |
| P value |  | **0.003 (0.08)** | **0.0001 (0.04)** | **0.0007 (0.02)** |
| iGAS | 28 | 18/252  7.1(4.5-11.1) | 18/166  10.8 (6.9-16.6) | 18/252  7.1 (4.5-11.1) |
| iGAS | 89 | 23/252  9.1(6.1-13.4) | 20/166  12.1 (7.9-18.0) | 23/252  9.1 (6.1-13.4) |
