# Supplementary material for: Genome analysis following a national increase in Scarlet Fever in England 2014
Source: BMC Genomics. 2017 Mar 10;18:224. doi: 10.1186/s12864-017-3603-z (PMC5345146; doi:10.1186/s12864-017-3603-z)

**Additional file 2.** Figure of *emm* gene type reference laboratory referrals from patients with SF fever from 2004-2014. *emm* gene type reference laboratory referrals from patients with SF fever from 2004-2014. Note this dataset is based on referred isolates only and is therefore biased: RVPBRU collate SF isolates routinely if part of localised cluster investigation and numbers of referred isolates for SF isolates are low (95% CI not included).


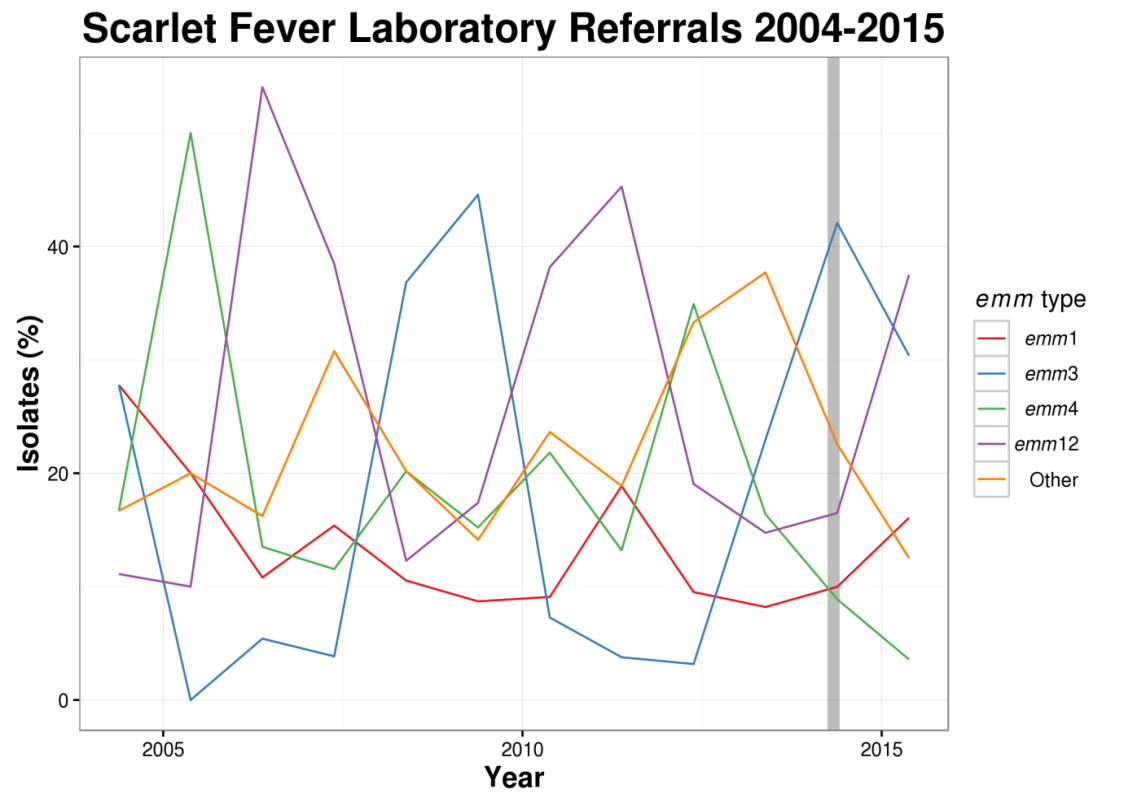

Supplement: Additional file 2: — Figure of emm gene type reference laboratory referrals from patients with SF fever from 2004–2014. emm gene type reference laboratory referrals from patients with SF fever from 2004–2014. Note this dataset is based on referred isolates only and is therefore biased: RVPBRU collate SF isolates routinely if part of localised cluster investigation and numbers of referred isolates for SF isolates are low (95% CI not included). (DOC 212 kb) [file 12864_2017_3603_MOESM2_ESM.doc]
