# Supplementary material for: Genome analysis following a national increase in Scarlet Fever in England 2014
Source: BMC Genomics. 2017 Mar 10;18:224. doi: 10.1186/s12864-017-3603-z (PMC5345146; doi:10.1186/s12864-017-3603-z)
Supplement: Additional file 3: — Table of isolate numbers per region. (DOC 44 kb) [file 12864_2017_3603_MOESM3_ESM.doc]

**Additional file 3.** Table of Isolate numbers per region

|  | Reported cases prior to commencement of isolate collation | Target number  of isolates | Received (6^th^ January -18^th^ June 2014) | Genomic sequence analysis | Representative strains from SNP clades | Normalised Random strains to 5% per region when possible |
| --- | --- | --- | --- | --- | --- | --- |
| Anglia & Essex | 318 | 13.4 | 2 | 2 | 2 | 2 |
| Avon, Glouc. & Wilts | 553 | 23.3 | 65 | 44 | 19 | 24 |
| Cheshire & Merseyside | 331 | 13.9 | 1 | 1 | 1 | 1 |
| Cumbria and Lancs | 305 | 12.8 | 107 | 75 | 21 | 13 |
| Devon, Cornwall & Somerset | 239 | 10 | 35 | 15 | 6 | 10 |
| East Midlands | 1166 | 49 | 51 | 40 | 13 | 40 |
| Greater Manchester | 318 | 13.4 | 11 | 8 | 8 | 8 |
| Kent, Surrey & Sussex | 478 | 20.1 | 16 | 13 | 9 | 13 |
| London | 684 | 28.8 | 31 | 25 | 13 | 25 |
| North East | 453 | 19 | 32 | 26 | 10 | 20 |
| South Midlands and Herts | 380 | 16 | 1 | 1 | 1 | 1 |
| Thames Valley | 404 | 17 | 0 | 0 | 0 | 0 |
| Wessex | 338 | 14.2 | 23 | 10 | 7 | 10 |
| West Midlands | 516 | 21.7 | 15 | 8 | 6 | 8 |
| Yorkshire & Humber | 652 | 27.4 | 40 | 35 | 16 | 27 |
| TOTAL | 7135 | 300 | 430 | 303 | 132 | 202 |
