# Supplementary material for: Genome analysis following a national increase in Scarlet Fever in England 2014
Source: BMC Genomics. 2017 Mar 10;18:224. doi: 10.1186/s12864-017-3603-z (PMC5345146; doi:10.1186/s12864-017-3603-z)
Supplement: Additional file 4: — Table of known virulence factors and antibiotic resistance determinants examined in genomes. (DOC 35 kb) [file 12864_2017_3603_MOESM4_ESM.doc]

**Additional file 4.** Table of known virulence factors and antibiotic resistance determinants examined in genomes

| ccpA_13 | emm1 | endoS_6 | Fba_10 | fbaA |
| --- | --- | --- | --- | --- |
| GRAB | gyrA | hasA_4 | hasB_11 | hasC_7 |
| ideS | LaCD | Mac_3 | mef | mefMB56 |
| Spyo029 | mefV1 | mefV2 | mefV3 | MF4 |
| MtsR_10 | nga_16 | parC | PerR | PrtS_13 |
| rgg_4 | ropB_12 | sagA_12 | SagB_10 | SagC_11 |
| scl1_orig | scl2 scpA_44 | sda1 | sda_orig | sic |
| ska | slo_2 | sof | Spd3_4 | Spd3_10 |
| Spd_4 | speA_1 | speA_6 | speB_3 | speC |
| SpeG | speJ | Spy1063 | Spy1064 | Spy1065 |
| spy1066 | Spy1438 | SpyA_9 | tetM_orig | tetO |
| ABC-transporter | CovR_9 | CovS_12 | Cpa | FctA |
| FctB_9 | mefA | MsmR | Nra | PrtF2 |
| srtC2 | UmuC-MucB | SSA |  |  |
