# Supplementary material for: Genome analysis following a national increase in Scarlet Fever in England 2014
Source: BMC Genomics. 2017 Mar 10;18:224. doi: 10.1186/s12864-017-3603-z (PMC5345146; doi:10.1186/s12864-017-3603-z)
Supplement: Additional file 5: — Table of 57 documented phage examined in genomes. (DOC 34 kb) [file 12864_2017_3603_MOESM5_ESM.doc]

**Additional file 5.** Table of 57 documented phage examined in genomes

| HKU360vir | HK360ssa | 315.1 | 315.2 | 315.3 |
| --- | --- | --- | --- | --- |
| 315.4 | 315.5 | 315.6 | 370.1 | 370.2 |
| 370.3 | 370.4 | Manfredo.1 | Manfredo.2 | Manfredo.3 |
| Manfredo.4 | MGAS10270.1 | MGAS10270.2 | MGAS10270.3 | MGAS10270.4 |
| MGAS10270.5 | MGAS10394.1 | MGAS10394.2 | MGAS10394.3 | MGAS10394.4 |
| MGAS10750.1 | MGAS10750.2 | MGAS10750.3 | MGAS10750.4 | MGAS2096.1 |
| MGAS2096.2 | MGAS5005.1 | MGAS5005.2 | MGAS5005.3 | MGAS6180.1 |
| MGAS6180.2 | MGAS6180.3 | MGAS6180.4 | MGAS8232.1 | MGAS8232.2 |
| MGAS8232.3 | MGAS8232.4 | MGAS9429.1 | MGAS9429.2 | MGAS9429.3 |
| NZ131.1 | NZ131.2 | NZ131.3 | NZ131.4 | SPsP1 |
| SPsP2 | SPsP3 | SPsP4 | SPsP5 | SPsP6 |
| UK-M3.1 |  |  |  |  |
